# Respiratory and Allergic Effects in Children Exposed to Pesticides—A Systematic Review

**DOI:** 10.3390/ijerph17082740

**Published:** 2020-04-16

**Authors:** Rafael Junqueira Buralli, Amana Freitas Dultra, Helena Ribeiro

**Affiliations:** 1 Departamento de Saúde Ambiental, Faculdade de Saúde Pública, Universidade de São Paulo, São Paulo-SP 01246-904, Brazil; amanadultra@gmail.com (A.F.D.); lena@usp.br (H.R.); 2 Department of Environmental and Occupational Health and Public Health Emergency Surveillance, Brazilian Ministry of Health (DSASTE/SVS/MS), Brasília-DF 70723-040, Brazil

**Keywords:** pesticide, child, lung function, respiratory symptoms, allergy

## Abstract

Pesticide exposure may affect children’s respiratory and allergic health, although results from epidemiological studies have not reached consensus. This review aims to analyze the scientific evidence on respiratory and allergic effects of exposure to agricultural pesticides in children aged up to 12 years old. The databases PubMed, Web of Science, Scielo, and Lilacs were screened to select articles published in English, Spanish, or Portuguese, and 21 articles were included in this review. Most investigations were conducted in North America (mostly in the United States), while no studies conducted in Latin America or Africa were found, despite their intensive use of pesticides. Children are exposed to pesticides through multiple pathways from the prenatal period throughout later developmental stages and may experience several respiratory effects. Most studies (79%) found positive associations with pesticide exposure and children’s respiratory and allergic effects such as asthma, wheezing, coughs, acute respiratory infections, hay fever, rhinitis, eczema, chronic phlegm, and lung function impairments. Contrastingly, 21% of the studies found no associations between pesticide exposure and children’s respiratory health. The vast differences among the characteristics of the studies hamper any comparison of the results. Exposure to pesticides may have several impacts on childhood respiratory health. More studies must be conducted, especially in low- and middle-income countries, preferably with comparable research protocols adapted to local realities. Efforts should be made to develop comprehensive risk mitigation strategies and behavioral interventions to reduce children’s exposure to pesticides used in agriculture and respiratory health effects, and to ensure healthy childhood growth.

## 1. Introduction

Epidemiological studies with adults suggest that occupational exposure to pesticides is associated with a higher prevalence of respiratory symptoms [1,2], asthma and allergies [3,4], and changes in lung function [1,2,5,6,7]. These findings were also reinforced by two literature reviews recently published [8,9]. However, little is known about the effects of exposure to pesticides used in agriculture on childhood respiratory health.

Children of farmers are at risk of pesticide exposure for multiple reasons, including: living close to agricultural fields; engaging in farm work from an early age; eating fruits and vegetables directly from the fields or soon after harvest; facing “take-home” exposure from farmworkers in their homes; being exposed during pesticide use at home for pest control [10,11]; and accidents with chemicals. Other factors may influence childhood exposure to pesticides, such as the mother’s exposure during pregnancy, time spent on the floor in areas of pesticide deposition, hand-to-mouth habits, and having diets often based on foods with a higher concentration of residues (e.g., fruits, juices and milk) [9,10,12]. Furthermore, children eat, drink and breathe more in proportion to personal weight than adults [10], have greater physiological susceptibility during development [13], and have a lower ability to metabolize and eliminate chemicals [14].

Few studies have examined associations between childhood exposure to pesticides and respiratory health, and the studies that have been carried out have not reached consensus. Maternal reporting of general pesticide exposure was associated with increased reports of chronic respiratory symptoms among school-aged children in Lebanon [15]. A case-control study found that children with reported pesticide exposure in the first year of life had 2.4 times the odds of developing asthma compared to those with no exposure [16]. Residential use of pesticides was weakly associated with respiratory symptoms among children under 18 years of age in the U.S. National Health and Nutrition Examination Survey (NHANES) [17]. However, a study in the Netherlands found no association between living near agricultural fields likely to be treated with pesticides and asthma and related respiratory symptoms [18]. 

Only restricted studies have examined the effects of specific pesticides on children’s respiratory health. Higher exposure to organophosphate pesticides (OP) during pregnancy and childhood (from birth to age five) were associated with increased odds of respiratory symptoms at age seven [19], and higher phosphate metabolites (diethyl and dimethyl) in childhood were associated with decreased lung function at seven years of age [20] among the children of farmworkers from California. Contrastingly, a cross-sectional study in NHANES found no associations of urinary OP metabolites (dialkyl phosphate and serum dichlorodiphenyldichloroethylene), and asthma risk [21]. A birth cohort study assessed pesticide exposure through personal air samples, and found that higher prenatal measurements of the pyrethroids cis-permethrin, but not trans-permethrin, were associated with increased cough symptoms in children by age five [22]. 

In order to verify the state of established knowledge regarding this important issue, a systematic review was undertaken to gather the published scientific evidence on the respiratory and allergic effects of children’s exposure to pesticides used in agriculture.

## 2. Materials and Methods 

This systematic review was conducted in accordance with the Preferred Reporting Items for Systematic Review and Meta-Analysis (PRISMA) process to identify published scientific evidence about respiratory and allergic effects in children exposed to agricultural pesticides. The PECO framework was used to delineate this review based on: (a) population: children up to 12 years old; (b) exposure: pesticides of all classes and types of use; (c) comparison: without the restriction of comparator groups (e.g., children not exposed to pesticides or exposed to different intensities and sources); (d) outcomes: All respiratory morbidities (e.g., symptoms, asthma, obstructive diseases) and lung function impairments.

The databases PubMed (www.ncbi.nlm.nih.gov/pubmed/), Web of Science (www.isiknowledge.com), Scielo (search.scielo.org), and Lilacs (pesquisa.bvsalud.org/portal/advanced/) were consulted to select articles in English, Spanish, or Portuguese using the following terms: For English: (pesticid* OR agrochemic* OR fumigant* OR fungicide* OR insecticide* OR herbicide* OR acaricide* OR nematicide*) AND (child* OR pregnan* OR prenatal OR offspring OR newborn OR early-life OR infant* OR preschool*) AND (respirat* OR pulmonar* OR asthma* OR allerg* OR hypersensitivit* OR rhinitis). For Spanish and Portuguese: (pesticid* OR plaguicida* OR agrotoxico* OR agroquimic* OR fumigant* OR fungicida* OR inseticida* OR insecticida* OR herbicida* OR acaricida* OR nematicida*) AND (niñ* OR criança* OR emabaraz* OR gravid* OR gesta* OR prenatal* OR pré-natal* OR hij* OR filh* OR nascid* OR infant* OR escolar*) AND (respirat* OR pulmon* OR asma* OR alerg* OR hipersensitivit* OR rinit*). 

The search on PubMed was conducted by the title, abstract, and MeSH terms; the search on Web of Science included topic (title, abstract and keywords); the search on Scielo included all indexes; and the search on Lilacs included all words. No temporal filter was established, and articles published until September 2019 were included in this review.

Only articles with full text available were included; and review, meta-analysis, experimental, qualitative and case studies were excluded. Articles focused exclusively on residential exposure to pesticides, and house allergens such as dust, cats, dogs, mites and cockroaches were also excluded. In the first screening, two independent reviewers read the article’s title, and selected those focusing on pesticide for agricultural use, being as inclusive as possible. In this screening, 163 articles were selected for the screening phase, and three reviewers independently read all abstracts to select relevant papers regarding the association of childhood respiratory health and agricultural pesticides. At this point, 136 articles were excluded because they did not meet the inclusion criteria or deal directly with the issue investigated. In case of disagreement, the three reviewers decided together what to do. Articles about respiratory and allergic effects of pesticide exposure in children from all ethnic groups, geographical location, and socioeconomic status were selected. Furthermore, each of the remaining 27 articles were fully read by at least two reviewers to gather the relevant information. Finally, the data extracted from the selected articles were checked for consistency by two reviewers. 

**Figure 1:**
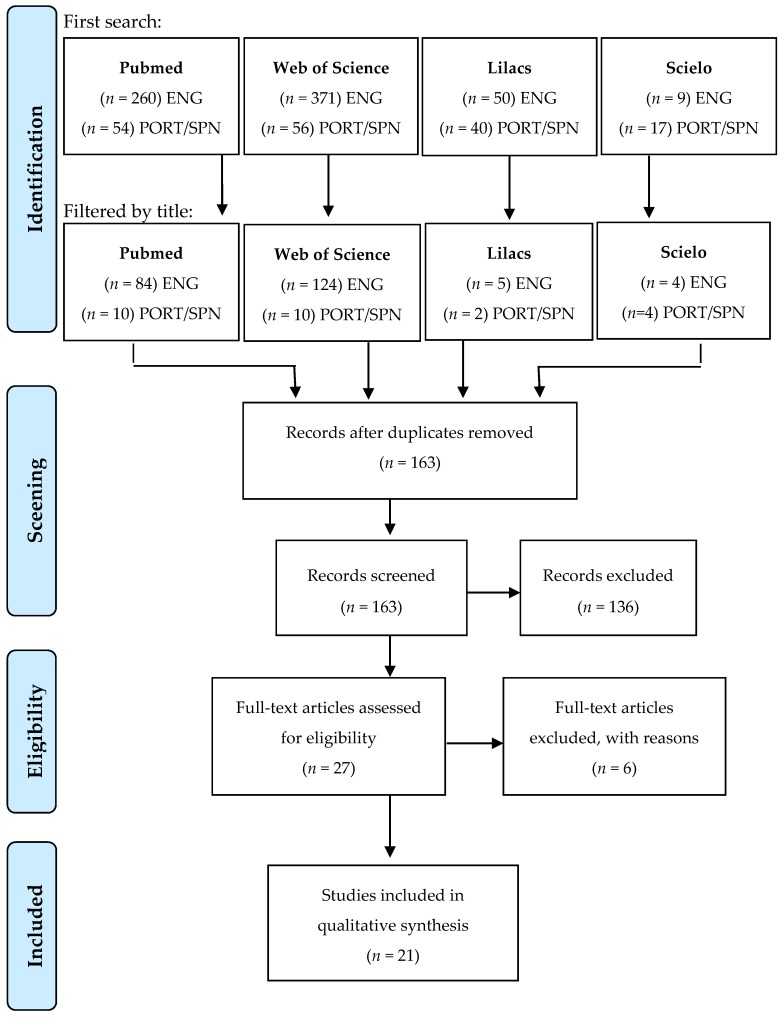
Flow chart of study selection.

After reading the full-text of articles considered eligible (27), six manuscripts were excluded for the following reasons: exposure study without addressing health effects; study on residential exposure to pesticides; study about house dust mite allergens; study with adults as subjects; and hospital-based studies which addressed differences between rural and urban populations; and children’s clinical course after pesticide poisoning without focusing on respiratory effects. Thus, 21 articles were finally selected and are discussed in this review (Figure 1).

The remaining selected articles were organized by the study location, design and year of publication, sample size and age, types of pesticides, exposure pathway and activity, exposure assessment method, period of exposure, health effect and assessment method, and main findings. These data were discussed, and study results compared, considering the prevalence, risk ratios, and odds ratios of health outcomes.

## 3. Results and Discussion

The studies included in this review are displayed in Table 1, Table 2 and Table 3, divided by the classes of pesticides assessed. Articles were separated in OP, OC, and multiple pesticides without assessing health effects separately (presented in Table 1, Table 2 and Table 3, respectively) because of their potential differences in characteristics, toxicity, and health effects on humans. 

Although OC has been banned in almost all countries, due to its long half-life it is still found in many human and environmental matrices. OC has been replaced by pesticides considered less persistent, but which were later shown to also be very toxic, such as OP, CM, and pyrethroids, among others [23]. Recent epidemiological studies have shown increasing evidence of the respiratory effects of pesticide exposure, raising concern for this public health issue. 

To date, only 21 epidemiological studies were indexed in the searched databases about the effects of agricultural pesticide exposure on childhood respiratory and allergic health. Of the 21 articles included in this review, some belonged to the same research project/program, resulting in 16 independent studies. Of these, 8 were cohort studies (7 prospective and 1 retrospective), 3 cross-sectional, 2 case-control, 2 hospital-based analyses of admissions and medical charts, and 1 retrospective survey. Five studies were about the CHAMACOS cohort study from University of California, Berkeley. 

**Figure 2:**
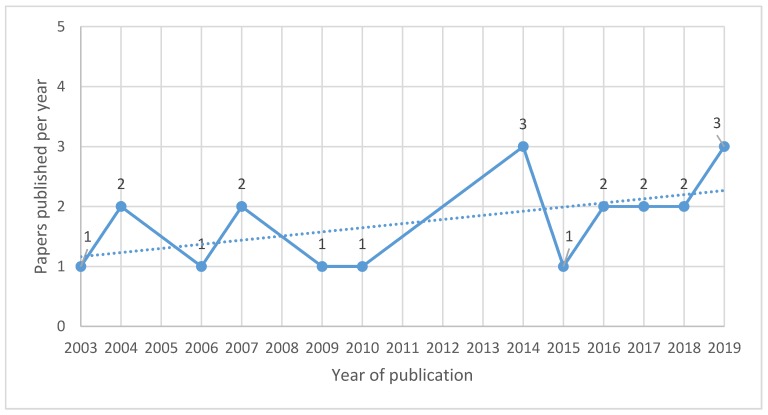
Number of papers published per year focused on children’s respiratory and allergic effects of pesticide exposure (*n* = 21).

The first study was published in 2003 and since then, there was a discrete upward tendency, with no publications during several years such as in 2005, 2008, and between 2011 and 2013 (Figure 2). 

All studies were located in three continents (North America, Asia and Europe), while Latin America and Africa had no studies, despite their intensive use of pesticides. A study conducted in Niger compared the respiratory effects on children from exposure to agricultural and pasture areas using questionnaires, but the results indicated that no farmer was using pesticides for agricultural purposes, only at home. Although an association between residential use of pesticides and cough without fever has been indicated among children, the article was not included in this review, which focuses solely on the agricultural use of pesticides [24]. As shown in Table 4, the majority of studies were conducted in the North American region, and more than half were from the United States.

Pesticide exposure may disproportionally affect lower and middle-income countries, where regulation, surveillance and farmer’s technical support are weaker and less rigorous [25,26]. In these countries, frequent exposure to multiple highly toxic chemicals without proper personal protection is common, and has been associated with respiratory health impacts in adults exposed occupationally [2,7,27,28,29], possibly overloading the health systems. It is estimated that 99% of the poisoning cases of pesticide exposure occur in low and middle-income countries [30], but contrastingly most studies included in this review were located in high-income countries. Although some children’s health effects of pesticide exposure have been studied recently in lower income countries [31,32,33], respiratory outcomes are still understudied. 

The study sample size and age of subjects varied widely among the 21 studies included in this review. The study with the least number of participants was a U.S. cohort that followed 16 children aged 6 to 16y (2 articles published), and the study that included the most participants followed a British birth cohort (ALSPAC) of 13,971 children aged up to 8.5y, and a U.S. cross-sectional population based study (NHANES) of 10,077 children aged 6 to 16y. Of all the studies, 33% (*n* = 7) reported childhood respiratory effects at specific ages (being one, two, and four studies respectively with children aged 1, 5, and 7 years), while 66% (*n* = 14) presented results of children of a wide age range, varying from newborns to 16 years old. These great differences in the studies’ sample sizes and subjects’ ages hamper the comparison of the results by specific age ranges. 

Children’s exposure to pesticides often begins before birth through the mother’s and father’s occupational exposure to pesticides, and tends to continue thereafter. A study of 260 farmworker mothers observed that all of them worked during pregnancy either entirely, or partially and have stopped working at week 22.8 ± 7.9 (mean, sd). They also reported that ~52% of the fathers worked in agriculture during pregnancy [34]. Twelve studies assessed respiratory effects considering prenatal exposure to pesticides, with only two of them focusing exclusively on prenatal exposure. One of them found that prenatal exposure to DDE (concentration at cord blood samples) was associated with wheezing at age 4y, but not thereafter, and with other respiratory outcomes at different ages [35]. Another study, which investigated children living on farms with pesticide use compared to children living on farms with no pesticide use, found that prenatal exposure to pesticides was associated with significantly higher odds of developing allergies and hay fever, but was not associated with coughs, bronchitis and asthma [36].

Children are exposed to pesticides through multiple pathways, which can include: having a mother exposed to pesticides while pregnant, living or studying near areas with pesticide use, attending agricultural areas, having parents working in agriculture, having residential exposure through take-home exposure, and eating and drinking contaminated food and water, among others [10,37]. A hospital-based multisite study in Sri Lanka observed that most children poisoned with pesticides had at least one parent engaged in farming activities (~58%), and were exposed at cultivation sites (~51%) [38]. 

Five out of the 21 studies included in this review used the parent’s occupational exposure to pesticides as a proxy for exposure. Of these, one study found that prenatal and childhood OP exposure (assessed by DAP metabolites) was non-significantly associated with respiratory symptoms at 5y and 7y [19], but was associated with significant decreases in lung function at age 7 in another study [20]. Prenatal exposure to pesticides (OP and pyrethroids) was shown to increase the risk of early cough, wheeze, and IgE production [22], and maternal postnatal occupational exposure to biocides/fungicides increased the likelihood of childhood wheeze [39]. Moreover, mothers working in ferneries were 2.7 times more likely to report a child’s diagnosis of a respiratory condition, compared to nursery farm workers [34]. While nursery farm workers are less exposed due to the lower height of the exposure, those working at ferneries apply pesticides at the same level as their breathing.

Four studies assessed the children’s respiratory effects from the residential proximity to pesticide application areas. Two studies found that asthma (observed through increased uLTE4 levels) was associated with OP exposure among children of farmers who live close to cultivation sites, with this association being significant only for diethyl alkyl phosphate (EDE) [40], even when PM 2.5 was considered as a joint exposure [41].

Childhood respiratory symptoms, asthma prevalence and pulmonary function changes were associated with elemental sulfur applications within 0.5 and 1 km radii of the residence [42], but non-significant associations were observed for residential proximity to fumigants used in the same cohort [43]. Furthermore, one study explored the children’s school proximity to the cultivation areas with pesticide use and respiratory effects, and found that urinary CM metabolite levels (by ETU concentration) were significantly associated with an increased risk of asthma and rhinitis [44].

Three other studies considered the very fact of children living in rural areas as a proxy for exposure. Associations between an increased prevalence of asthma and wheeze, and elevated Th2 levels among children exposed to OP at early ages in the U.S. were observed [45]; there were also significantly higher odds of allergies and hay fever in Canadian children who lived on farms with pesticide use during pregnancy, compared to children living on farms with no pesticide use [36]; and a higher prevalence of respiratory symptoms such as wheezing, allergic rhinitis, and eczema were found among rural children from conventional tea plantations compared to children from an organic tea cultivation in Sri Lanka [46].

Ten out of the 21 studies (47.7%) assessed the health effects of combined exposure to more than one pesticide, including OP, carbamates (CM), pyrethroids, herbicides, fungicides and biocides. Moreover, five studies (23.8%) addressed the specific effects of OP, four (19.0%) investigated exposure to organochlorines (OC), mainly DDT and its main metabolite DDE, while the two remaining studies focused on exposure to elemental sulfur and fumigants (one each). 

Exposure to OP was significantly associated with asthma and wheeze in children at early ages [45], decreases in lung function at 7 years old [20], and increased chances of developing asthma [40,41]. The combined exposure including OP (with pyrethroids) was related to higher risk of early cough, wheeze, and IgE as a biomarker for allergies [22], while no associations were found between asthma and biomarkers of DAP or DDT insecticides among 10,077 school aged children in a population-based study in the U.S. [21]. Contrastingly, prenatal and childhood exposure to OP through DAP metabolites were not associated with respiratory symptoms at 5y and 7y [19].

Prenatal exposure to OC was associated with respiratory tract infections early in life among 199 Inuit infants, a population that is mostly exposed through fish and mammal fat consumption [47]. In another study, prenatal exposure to OC was associated with wheeze at age 4y, but not thereafter, and other respiratory outcomes at different ages up to 14 years old [35]. In a hospital-based case control study with 620 Chinese children aged 3 to 6 years, asthmatic children presented significantly higher levels than non-asthmatic children of seven OC chemicals (α-HCH, HCB, β-HCH, γ-HCH, Heptachlor, p,p’-DDE and o,p’-DDT) [48]. A study of 747 boys aged 12 to 21 months in Mexico investigated the effects of pesticides applied for malaria control in maternal serum samples, and found no associations with lower respiratory tract infections such as pneumonia and bronchiolitis [49].

Eight studies addressed the childhood respiratory effects of multiple pesticides combined through questionnaires and interviews, and observed significantly higher odds of developing asthma, wheezing, and chronic phlegm [15], and also wheezing, allergic rhinitis and eczema [46], when compared with non-exposed children. Maternal postnatal exposure to biocides and fungicides at work was associated with an increased risk of childhood wheeze [39]. Asthma diagnosis before 5 years old was also associated with exposure in the first year of life to herbicides and other pesticides [16]. 

CM exposure (measured through urinary ETU) in a school located in a vineyard area with high pesticide use was significantly associated with an increased risk of asthma and rhinitis, but not of lung function changes [44]. 

Two studies had no clear associations between exposure to pesticides and health effects, with one of them being a comparison between ferneries and nursery farm workers [34], and the other a multisite hospital-based study assessing the prevalence of respiratory symptoms among poisoned children [38]. The former presents differences on regarding the type of activities regarding plants with different morphologies. While nursery farm workers are less exposed due to the lower height of the exposure, those working at ferneries apply pesticides at the same level of their breathing. The later reported respiratory symptoms in about 8.6%–10.8% of poisoned children, and most had at least one parent engaged in farming activities [38].

Finally, 15 out of the 19 remaining studies (79%) included in this review found positive associations between pesticide exposure and childhood respiratory and allergic effects, mainly asthma [15,16,40,41,42,45,48], wheezing [15,22,39,45,46], allergic rhinitis and eczema [46], coughing and allergies [22], respiratory acute infections [47], chronic phlegm [15], and lung function impairments [20]. 

Prenatal exposure to DDE was associated with children’s respiratory outcomes at different ages, and with wheeze at age 4, but not thereafter [35]. Significantly higher odds of allergies and hay fever were observed among children who lived on farms with pesticide use during pregnancy, compared to children living on farms with no pesticide use, but no relation was observed for cough, bronchitis and asthma [36]. Urinary ETU levels (as biomarkers for CM) were significantly associated with an increased risk of asthma and rhinitis, after adjustment, but no significant associations were found between lung function changes [44].

Contrastingly, four studies out of the 19 remaining studies (21%) found no associations between exposure to pesticides and respiratory or allergic health effects. Prenatal and childhood exposure to OP (assessed through urinary DAP) were non-significantly associated with respiratory symptoms at ages 5 and 7 years old [19]. Higher prenatal exposure to OC (*p,p*-DDE and *p,p*-DDT in maternal serum) was not associated with a higher risk of lower respiratory tract infections [49]. 

A population-based study in U.S. did not observe clear associations between asthma and OP and OC exposure (which was measured using biomarkers of DAP or DDT insecticides) among school-aged children [21]. No significant associations were observed between residential proximity to fumigant use during pregnancy and from birth to age 7 (assessed through the pesticide use registration in California) and respiratory symptoms, use of asthma medication, and lung function measurements [43].

The correlation of respiratory and allergic effects between the studies included in this review was compromised by the considerable variability in the studies’ characteristics (or lack of information), which represents a risk of bias for comparison across studies. For instance, the studies’ differences were related to a) study and participants: study design, health outcomes, and health assessment methods, number and characteristics of participants (e.g., age range and sex), and follow-up period; and b) exposure: types of pesticides, quantity, frequency and methods of utilization, exposure pathway, and assessment method. Despite these limitations, this review presents relevant findings to help understand how children are exposed to agricultural pesticides and the respiratory and allergic effects of such exposure.

## 4. Conclusions

This comprehensive review gathered published evidence on the respiratory and allergic effects of agricultural pesticide exposure in children. It shows that children have been exposed to pesticides through multiple sources and pathways from the prenatal period throughout early development, and this may have several respiratory effects such as asthma, wheezing, cough, allergies, and pulmonary function impairment. Although environmental exposure to pesticides has been associated with an increased risk of respiratory outcomes among children, the causal relationship is still under debate, and more scientific evidence must be produced.

Children from low- and middle-income countries are probably the most affected due to excessive use of highly toxic pesticides, lack of occupational training and protective measures, and high environmental and take-home exposure, among other factors [10,25]. However, most studies are still being conducted in high-income countries. Therefore, this review highlights the necessity of more studies in low- and middle-income countries, especially those with larger food production, which may employ more conventional methods of pesticide use and have large child populations in rural areas and helping in agricultural work. Furthermore, novel methods have been used to explore the health effects of environmental contaminants, such as metabolomics, which may be valuable to advance the current knowledge of the mechanisms of respiratory and allergic effects.

In this study, the comparison of the effects of exposure to pesticides was hampered by study differences, such as the types of pesticide investigated, exposure pathway and assessment method, and age range, among others. The development and validation of international research protocols, adapted to local specificities, should help to develop comparable studies in different settings, facilitating a better understanding of the complexities of pesticide exposure and resulting health effects. 

Despite its limitations, this review provides important epidemiological evidence that reinforces the relevance of strengthening public policies to protect children’s health. We recommend additional research to fully characterize childhood exposure pathways to pesticides and the links with respiratory health outcomes. Moreover, efforts should be made globally in order to develop more comprehensive risk mitigation strategies and behavioral interventions based on the current information, seeking to minimize children’s exposure to agricultural pesticides and respiratory health effects.

## Figures and Tables

**Table 1:** Studies on respiratory and allergic effects of organophosphate pesticides (OP) in children.

Author, Year and Country	Study Design	Study Sample	Pesticides Addressed	Exposure Pathway and Activity	Exposure Assessment Method	Period of Exposure	Health Effect and Assessment Method	Results
Duramad, P. et al. 2006/USA	Longitudinal birth cohort (CHAMACOS)	412 children aged up to 24 months	OP	Children who reside in agricultural settings	Questionnaire-based interviews with home visits	Prenatal up to 24 months	Respiratory symptoms through questionnaire-based interviews, medical records, and Th1 and Th2 cytokines as biomarkers of allergic asthma.	Asthma and wheeze outcomes in children at 24 months of age were associated with elevated Th2 status in children at early age.
Raanan, R. et al. 2015/USA	Longitudinal birth cohort (CHAMACOS)	364 children aged 5y and 7y	OP	At least 1 agriculture worker in household	Maternal interviews and urinary DAP	Prenatal and childhood until age 5y and 7y	Respiratory symptoms through questionnaire (ISAAC)	Higher prenatal and childhood DAP [] were non-significantly associated with respiratory symptoms at 5y and 7y.
Raanan, R. et al. 2016/USA	Longitudinal birth cohort (CHAMACOS)	279 children aged 7y	OP	At least 1 agriculture worker in household	Maternal interviews, home visits, six urinary DAP measured during pregnancy and five times in childhood	Prenatal and childhood until 7y	Spirometry	Childhood diethyl, dimethyl and total DAP [] were associated with significant decreases in lung function at age 7. Significantly lower FEV_1_ (ß = −0.16, 95%CI: −0.30; −0.02), and FVC (ß = −0.17, 95% CI: −0.34; 0.01) were observed per 10-fold increase of children’s total DAP levels.
Benka-Coker, WO. et al. 2019a/USA	Longitudinal cohort with 4 months follow-up	16 asthmatic children aged 6 to 16y from agricultural communities	OP and joint effects with PM2.5 and Ozone	Residential proximity to crop areas, and parents involvement in agriculture	Repetitive urine samples for DAP (summative measures), and PM2.5 and Ozone [] data from local monitoring stations	Not clear	Asthma assessed through urinary uLTE4 collected every 6 days during the study period	Higher exposures to OP were associated with increases in the LTE4 levels, and concurrent short-term exposure to PM 2.5 was associated with an increase in a marker of asthma morbidity.
Benka-Coker, WO. et al. 2019b/USA	Longitudinal cohort with 4 months follow-up	16 asthmatic children aged 6–16y from agricultural communities	OP	Residential proximity to crop areas, and parents’ involvement in agriculture	Repetitive urine samples for DAP, and comparison with another population-based cohort (NHANES). The total OP exposure was estimated through summative DAP measures, rather than individual measures	Not clear	Asthma assessed through urinary LTE4 collected every 6 days during the study period	Distribution of summed DAPs in this study were significantly higher than NHANES levels. Increase in uLTE4 levels were associated with increased exposures to DAPs, being significant only for EDE levels, after adjustment: 8.7 (95%CI: 2.8, 14.6).

**Notes:** OP = organophosphate pesticides; Th1 = T-helper 1; Th2 = T-helper 2; DAP = dialkyl phosphate metabolites; [] = concentrations; FEV_1_ = forced expiratory volume in first second; CI = confidence interval; FVC = Forced vital capacity; PM_2.5_ = particulate matter <2.5 μg; LTE4 = Leukotriene E4; uLTE4 = urinary Leukotriene E4; EDE = diethyl alkyl phosphate.

**Table 2:** Studies on respiratory and allergic effects of organochlorine pesticides (OC) in children.

Author, Year and Country	Study Sample	Study Design	Pesticides Addressed	Exposure Pathway and Activity	Exposure Assessment Method	Period of Exposure	Health Effect and Assessment Method	Results
Dallaire, F. et al. 2004/Canada	199 Inuit infants up to 12 months	Review of medical charts	DDE	Environmental exposure, especially the consumption of fish and marine mammal fat	Maternal plasma during delivery and infant plasma at 7 months of age	Prenatal and at infancy	Comparison of incidence rates of upper and lower respiratory tract infections (URTIs and LRTIs, respectively) in two follow-ups (6 and 12 months)	Compared to rates for infants in the first quartile of DDE exposure (least exposed), adjusted RR for infants in higher quartiles ranged between 1.15 and 1.56 for URTIs, and 0.96 and 1.40 for LRTIs at the 6 months follow-up, while it ranged from 1.09 to 1.34 for URTIs, and from 0.98 to 1.13 for LRTIs at the 12 months follow-up, suggesting a possible association between prenatal exposure to OCs and acute infections early in life. Despite most RR were > 1.0, only URTIs at the 2^nd^ quartile were statistically significant at both follow-up.
Gascon M., et al. 2007/Spain	405 children aged up to 14y	Longitudinal birth cohort	DDE	Non-specific environmental exposure	DDE [] at cord blood samples, and immune biomarkers at age 4y	Prenatal exposure to DDE	Occurrence of wheeze, chest infections and asthma through questionnaire-based interviews at years 1, 2, 3, 4, 6.5, 10 and 14y	Prenatal DDE exposure was associated with wheeze at age 4y (RR per double of [] = 1.35; 95%CI: 1.07; 1.71), but not thereafter. Prenatal exposure was associated with children’s respiratory outcomes at different ages, but no associations were found between the immune biomarkers and DDE.
Cupul-Uicab, L.A. et al. 2014/Mexico	747 newborn singleton boys	Longitudinal birth cohort	DDE and DDT	Chiapas where DDT was applied in crops until 1991 and for malaria control until 1998	Maternal serum samples, and home visits	Prenatal exposure and from 12 months to 21 months	Mothers report on doctor diagnosis of pneumonia, bronchitis or other illness such as LRTI	Higher prenatal exposure to p,p-DDE and p,p-DDT were not associated with higher risk of LRTI before or after adjustment for confounders.
Meng, G. et al. 2016/China	620 asthmatic children, and 218 non-asthmatic children aged 3–6y	Hospital-based Case-Control	OC	Environmental exposure, mainly from agriculture and residential use	Self-completed questionnaire	Not clear	Physician-diagnosed asthma by questionnaire and spirometry, and questionnaire to assess allergy symptoms (rhinitis and eczema)	Asthmatic children presented significantly higher levels of 7 OC pesticides (α-HCH, HCB, β-HCH, γ-HCH, Heptachlor, p,p’-DDE and o,p’-DDT) than non-asthmatic children.

**Notes:** OC = organochlorine pesticides; DDE = dichlorodiphenyldichloroethylene; URTIs = upper respiratory tract infections; LRTIs = lower respiratory tract infections; RR = relative risk; CI = confidence intervals; [] = concentrations; DDT = dichlorodiphenyltrichloroethane; p,p-DDE, and p,p-DDT and o,p’-DDT = metabolites of DDE and DDT, respectively; α-HCH, β-HCH and γ-HCH = metabolites of hexachlorocyclohexanes; HCB = hexachlorobenzene.

**Table 3:** Studies on respiratory and allergic effects of multiple pesticides, elemental sulfur, and fumigants in children.

Author, Year and Country	Study Sample	Study Design	Pesticides Addressed	Exposure Pathway and Activity	Exposure Assessment Method	Period of Exposure	Health Effect and Assessment Method	Results
Salameh, PR. et al. 2003/Lebanon	3291 children aged 5–16y	Cross-sectional	Multiple pesticides	Environmental exposure of children from a randomly selected sample of public schools	Standardized questionnaire and residential exposure score, based on residential, para-occupational and domestic exposures	Not clear	Respiratory symptoms assessed by using the American Thoracic Society (ATS) questionnaire	Compared to non-exposed children, those exposed to pesticides had significantly higher odds of having respiratory disease (OR = 1.71; 95%CI: 1.20–2.43), asthma (OR = 1.73; 95%CI: 1.02–2.97), chronic phlegm (OR = 1.90; 95%CI: 1.26–2.87), recurrent wheezing (OR = 2.10; 95%CI: 1.39–3.18), and ever wheezing (OR = 1.99; 95%CI: 1.43–2.78), after adjustment for potential confounders.
Salam, MT. et al. 2004/EUA	4000 school-aged children in 12 Southern California	Case-control	Multiple herbicides and pesticides	Children who had early-life environmental exposure to pesticides and other contaminants	Questionnaire and telephone interviews with mothers to collect additional exposure and asthma history	Prenatal and at first year of life	Physician-diagnosed asthma by age 5, and controls were asthma-free at study entry, frequency-matched on age, sex, and local of residence	Asthma diagnosis before 5y was significantly associated with pesticide exposure in the first year of life to herbicides (OR = 4.58; 95% CI, 1.36–15.43), and pesticides (OR = 2.39; 95% CI, 1.17–4.89).
Weselak, M. et al. 2007/Canada	3405 children aged 0–12y or more, from family farmers	Retrospective cohort	Multiple pesticides	Living on a family farm	Questionnaires on pesticide use and involvement in agricultural activities	Prenatal	Respiratory symptoms through a questionnaire about health status	The odds of allergies and hay fever were significantly elevated in children who lived on farms with reported use of any pesticide, herbicides, fungicides, insecticides, OP, phenoxy, and 2,4-D during the pregnancy period, compared to children living on farms with no pesticide use. No associations were observed for persistent cough, bronchitis and asthma among the offspring of farm families.
Reardon AM. et al. 2009/EUA	652 children aged 5y	Prospective birth cohort study	OP (chlorpyrifos and diazinon), and pyrethroids (cis-permethrin and trans-permethrin)	Elementary school children in California having a mother working in agriculture	Retrospective questionnaire, and measurement of prenatal levels of pesticide	Prenatal and first year of life	Questionnaire about wheeze and analysis of IgE production (5y); increased levels of TH2 cytokines in children (2y)	Prenatal exposures to pesticides may influence the risk of early cough, wheeze, and IgE production. Individual pesticides may differ in regard to risk.
Tagiyeva, G. et al. 2010/England	13,971 children aged up to 102 months	Birth cohort-Avon Longitudinal Study of Parents and Children (ALSPAC)	Biocides and fungicides	Parental occupational exposure to pesticides, along with other contaminants	Questionnaire and clinic evaluations	Up to 102 months	Questionnaire, clinical assessments	Maternal postnatal occupational exposure to biocide/fungicide increased the likelihood of childhood wheeze (OR = 1.22; 95%CI: 1.02–2.05).
Perla, ME. et al. 2014/USA	10,077 children aged from 6y to 16y	Cross-sectional population-based (NHANES)	Survey questionnaires and exposure biomarkers for OP (DAP) and DDT	Environmental exposures	Blood and urine tests	Up to 16 years old	Questionnaire, blood and urine tests	No clear associations between asthma and biomarkers of DAP or DDT insecticides among school aged children in the USA. Exposure to DAP and DDT is widespread and variable in U.S. children but higher metabolite levels were observed among Mexican Americans.
Runkle, J. et al. 2014/USA	Snowball sample with 170 farmworker mothers (mostly from Mexico, but also indigenous from Central America and others)	Cross-sectional survey with participatory approach(CBPR)	Multiple pesticides	Mother’s involvement in agriculture at nurseries (n = 62) and ferneries (n = 108), pesticide handling activities, father’s work as a farmer	Interviews about current and past work conditions (e.g., duration of work in agriculture while pregnant, father’s work in agriculture)	Prenatal until 1y (~52% worked the entire pregnancy period and ~48% worked partially)	Interview about outcomes during last pregnancy, infant’s health for the first year of life and doctor-diagnosed respiratory and breathing problems	Most self-reported child health problems were respiratory-related (~76%). Significantly more mothers working in ferneries reported child diagnosis of any health problem and were 2.7 times more likely to report child diagnosis of respiratory condition, compared to nursery workers.
Raanan, R. et al. 2017/USA	347 children aged 7y living in an agricultural community	Longitudinal birth cohort (CHAMACOS)	Elemental sulfur	Residential proximity to agricultural areas with elemental sulfur applications	Sulfur application within 0.5, 1, and 3km of residences during the week, month, and 12 months prior to pulmonary evaluation using California’s Pesticide Use Report (PUR) data	Short-term exposure (week, month and a year) before the pulmonary assessment	Respiratory symptoms through questionnaires (ISAAC) with mothers, and spirometry with children at 7y	Sulfur applications within 0.5 and 1 km radii were associated with respiratory outcomes. Asthma medication use [OR = 3:51; 95% CI: 1.50; 8.23] and respiratory symptoms [OR = 2:09; 95% CI: 1.27; 3.46] significantly increased (both *p* = 0.004) and FEV_1_ significantly decreased (*b* = −0.14; 95% CI: −0:248, −0:039; *p* = 0:008) per 10-fold increase in the estimated amount of sulfur used within 1km of the residence during the year before pulmonary assessment.
Dayasiri, KC. et al. 2017/Sri Lanka	155 children aged 9 months to 12y with pesticide poisoning	Retrospective and prospective hospital-based multisite study (admissions from 2007 to 2014)	Most frequent: OP (41%), CM (23%) and herbicides (12%)	51.3% were exposed at cultivation sites, 18.9% at home gardens, and 16.2% at home kitchens	Questionnaire-based interviews in hospital admission	Not clear	Prevalence of respiratory symptoms among poisoned children, along with other symptoms	~74% had less than 5y. About 58% of children had at least one parent engaged in farming activities and 50% use pesticide in farming. Only 8.6%–10.8% (depending on which hospital) of poisoned children presented respiratory symptoms.
Kudagammana, ST.; Mohotti, K. 2018/Sri Lanka	182 preschool children aged 1–5y from an organic area (*n* = 81) and conventional area (*n* = 101)	Cross-sectional	Multiple pesticides	Children living close to tea plantations (conventional vs. organic)	Children from a conventional tea plantation were compared to children from an organic tea cultivation area	Not clear	Prevalence of allergic diseases through a questionnaire-based interview using the modified International Study of Asthma and allergies (ISAAC)	Wheezing was noted in 41.2% of children from the organic estate and 59.8% from the conventional estate. The respective percentages for allergic rhinitis were as 37.7% and 82.5% while for eczema they were 17.5% and 20.3%.
Gunier, RB. et al. 2018/USA	294 children living in an agricultural area	Longitudinal birth cohort CHAMACOS	Fumigants (methyl bromide, chloropicrin, metam sodium and 1,3-dichloropropene)	Residential proximity to agricultural areas using fumigants	Fumigant use within 3, 5 and 8 km of residences during pregnancy and from birth to age 7 using PUR data	Prenatal and from cumulative exposure from birth to 7y	Respiratory symptoms (ISAAC) and asthma medication use through questionnaires with mothers, and spirometry with children at 7y	No significant associations were observed between residential proximity to fumigants use and respiratory symptoms, use of asthma medication, and lung function measurements.
Raherison, C. et al. 2019/France	281 children aged 3–10y from 4 rural schools of a French vineyard region	Cross-sectional with two phases: during winter with no pesticide exposure, and summer, when pesticides are regularly applied	Multiple pesticides (insecticides, herbicides and fungicides) in outdoor air around schools, and phthalimides and dithiocarbamate fungicides in urine (ETU)	Proximity of schools from the edge of vineyards	Mobile stations located close to the schools for air monitoring, and urine measurements in a subset (*n* = 96) of studied children	Not clear	Asthma and rhinitis through ISAAC, and respiratory symptoms. A symptom score was made to classify children into low, moderate and high scores. Bronchial obstruction was measured to assess the FEV_1_ and FEV_6_ of the FVC, and spirometry was performed among children aged over 6y	12 pesticides were detected in the schoolyards (89% fungicides and 11% insecticides), and significantly higher values of urine ETU [] were observed in the higher pesticide use period (*p* = 0.012). Urinary ETU [] was significantly associated with an increased risk of asthma and rhinitis (OR: 3.6 [1.04–12.1], after adjustment. No significant associations were found between lung function changes and pesticide exposure in air, or urinary ETU.

**Notes:** OR = odds ratio; CI = confidence intervals; OP = organophosphate pesticides; 2,4-D = 2,4-Dichlorophenoxyacetic acid; IgE = Immunoglobulin E; TH-2 = T helper cells type 2; DAP = dialkyl phosphate metabolites; DDT = dichlorodiphenyltrichloroethane; CBPR = community-based participatory research; FEV_1_ and FEV_6_ = forced expiratory volume in first and sixth second, respectively; CM = carbamate pesticides; PUR = Pesticide Use Report; ISAAC = International Study of Asthma and Allergies in Childhood; ETU = ethylenethiourea; FVC = Forced vital capacity; [] = concentrations.

**Table 4:** Regional distribution of the articles considered in the present study.

Region	Number of Articles	%	Countries (*n*, %)
Asia	4	19.0	Sri Lanka (*n* = 2, 9.5%); Lebanon and China (*n* = 1 each, 4.8%)
Europe	3	14.3	France, England, and Spain (*n* = 1 each, 4.8%)
North America	14	66.7	United States (*n* = 11, 52.4%); Canada (*n* = 2, 9.5%); Mexico (*n* = 1, 4.8%)
Total	21	100	10 countries in total

**Note:** There were no studies conducted in Africa nor in South America.
